# The Influence of *Urtica dioica* and *Vitis vinifera* Fibers on the Thermal Properties and Flammability of Polylactide Composites

**DOI:** 10.3390/ma17061256

**Published:** 2024-03-08

**Authors:** Tomasz M. Majka, Radosław Piech, Marcin Piechaczek, Krzysztof Adam Ostrowski

**Affiliations:** 1 Department of Chemistry and Technology of Polymers, Faculty of Chemical Engineering and Technology, Cracow University of Technology, Warszawska 24, 31-155 Cracow, Poland; 2 Faculty of Civil Engineering, Cracow University of Technology, Warszawska 24, 31-155 Cracow, Poland

**Keywords:** natural fibers, biopolymer, biocomposites, flammability, thermal properties, polylactide

## Abstract

This study focuses on examining the influence of bast fibers on the flammability and thermal properties of the polylactide matrix (PLA). For this purpose, *Urtica dioica* and *Vitis vinifera* fibers were subjected to two types of modifications: mercerization in NaOH solution (M1 route) and encapsulation in an organic PLA solution (M2 route). In a further step, PLA composites containing 5, 10, and 15 wt% of unmodified and chemically treated fibers were obtained. The results of the tests show that only biocomposites containing mercerized fibers had a nearly 20% reduced flammability compared to that of PLA. Moreover, the biofiller obtained in this way belongs to the group of flame retardants that generate char residue during combustion, which was also confirmed by TGA tests. The M2 modification route allowed to achieve higher mass viscosity than the addition of unmodified and M1-modified fibers. The reason is that fibers additionally encapsulated in a polymer layer impede the mobility of the chain segments. The inferior homogenization of the M2-modified fibers in the PLA matrix translated into a longer combustion time and only a 15% reduction in flammability.

## 1. Introduction

The participation of fiber-reinforced polymer composites in the global industry is growing year-by-year. This is evidenced by current forecasts regarding the projected value of the global fiber-reinforced polymer (FRP) composite market. It is anticipated that the value of this market will increase by over 60% by 2029 compared to its value in 2022 [1,2]. The increasing interest in polymer composites stems from the benefits derived from their properties, such as high tensile strength, a high Young’s modulus, a favorable strength-to-weight ratio, high resistance to chemical corrosion, and ease of assembly [3].

However, an important factor affecting the polymer composites industry is the guidelines currently being introduced that include carbon footprint reduction and closed-loop principles [4]. Current methods of processing polymers reinforced with synthetic fibers lead to a significant reduction in the strength of the recovered fibers, up to 90% [5]. Therefore, in recent years, there has been a noticeable trend of promoting natural fibers and biopolymers [6].

Natural fibers are composed mainly of organic compounds which undergo thermo-oxidative degradation at temperatures above 180 °C. For this reason, thermoplastic polymers such as poly(ethylene terephthalate) are mainly used as matrices for polymer composites with natural fibers. Such a polyester matrix has an appropriate processing temperature range that does not negatively affect the properties of natural fibers [7,8,9,10,11,12].

Natural fibers can be divided into two groups: primary plant fibers (the main purpose of growing such plants is to obtain fibers from them) and secondary plant fibers (fibers are obtained as waste). Examples of primary plants are jute, kenaf, hemp, and sisal. Secondary plants include coconut, pineapple, and oil palm. Kenaf and jute fibers are the leaders in the worldwide production of natural fibers used in composites. Natural fibers could be also classified into six basic types: fibers from leaves (pineapple, sisal, manila), fibers from seeds (kapok, cotton, coconut), fibers from stems (hemp, kenaf, jute), bast fibers (kenaf, ramie, jute), grass and reed fibers (corn, wheat, rice), and all others (roots and wood) [13,14,15,16,17,18,19].

Natural fibers are composed of cellulose, hemicellulose, lignin, aromatic compounds, waxes, lipids, and water-soluble compounds. The content of individual ingredients depends on the type of natural fiber [20,21,22,23].

Cellulose in plant cell walls is in the form of microfibrils arranged parallel to each other, which ensures the stiffness and stability of the fiber. Compared to cellulose, hemicellulose is not chemically homogeneous and has a lower molecular weight. Since only some of the components of hemicellulose have a crystalline structure, it is susceptible to acidic and enzymatic hydrolysis processes. Natural fibers also contain lignin and aromatic compounds. Lignin is a polyphenylpropanoid complex that is formed from one or more alcohols. For this reason, we distinguish different types of lignin. Lignin in natural fibers is responsible for strength and stiffness [16,20,21,24].

Some cellulose areas are connected with fragments of lignin and hemicellulose. The angle of inclination of the cellulose microfibers to the fiber axis affects the physicochemical properties of the entire composite [25,26,27,28,29].

The use of natural fibers in polymer composites significantly reduces energy consumption over the entire life of the material compared to a polymer composite reinforced with synthetic fibers. Products made of polymer composites with plant fibers are much lighter, which is of great importance in the automotive industry.

As part of the conducted research, fibers from flexible stems of *Urtica dioica* and woody shoots of *Vitis vinifera* were utilized. Using the Scopus database, the results of previous research on fibers from *Urtica dioica* and *Vitis vinifera* were analyzed (Table 1). Some of the articles describe the use of these fibers as reinforcement in the production of polymer composites. Research on the strength parameters of nettle fibers has been ongoing for over 15 years [30]. However, the concept of utilizing fibers from *Urtica dioica* and *Vitis vinifera* in the production of polymer composites is less than 10 years old.

Fischer et al. [31] conducted tensile and flexural strength tests on polymer composites with nettle fibers ranging from 20% to 40% by weight. The fibers were not subjected to modification. PLA and PP were used as the base. The highest reinforcement effect was achieved for samples with 30% nettle fibers. A 12% increase in tensile strength and a 60% increase in Young’s modulus compared to the reference sample were obtained. Interestingly, in the case of 30% nettle fibers based on PP, a decrease in tensile strength and modulus of elasticity compared to the reference sample was observed. A similar relationship was also noted by Paukszta et al. [32].

Based on the above research results, Bogard et al. [33] conducted a study on a PLA-based polymer composite in which 10% by weight of prepared nettle fibers were used. Standard-shaped paddle samples were then prepared and tested for tensile strength. The obtained values of the modulus of elasticity and tensile strength were compared with the results presented in studies [34,35,36]. The authors pointed out the high scatter of values due to the parameters of the plant itself. On this basis, the authors pointed to the potential of using the fibers to produce composite polymers with low material strain.

Suarsana et al. [37], on the other hand, conducted research on an epoxy resin-based composite with modified nettle fibers. Some fibers underwent a silane chemical treatment that removed lignin and hemicellulose from the fiber while strengthening the matrix–fiber adhesion. The results showed that the composition ratio of nettle fibers to epoxy resin matrix and silane treatment had a significant impact on increasing the flexural strength compared to the reference sample.

Polymer composites with natural fibers based on a biodegradable matrix are also very popular. Such polymers include polylactide, polyglycolide, polyhydroxyalkanoates, and polycaprolactone [38,39,40,41,42,43,44,45]. The properties of composites with natural fibers are influenced by the type of ingredients used, the selection of proportions between the biopolymer matrix and the filler used, and the type of interactions between the hydrophilic phase and the hydrophobic phase. To obtain the best possible properties of such composites, fibers are modified [46,47,48] by chemical or physical treatment, leading to improvement mainly in thermal properties [49,50,51,52].

Research confirming the validity of using natural fibers as reinforcement to improve the thermal properties of polymer composites has been documented in numerous industry publications, including studies [53,54,55].

Chandramohan et al. [56] presented a study on epoxy resin reinforced with fibers from vetiver grass. To enhance the bonding between natural fibers and the matrix, the fibers underwent chemical treatment using NaOH. Subsequently, the composite was tested for tensile strength, dynamic analysis, and thermogravimetric analysis. The results indicated an increase in tensile strength for samples with 20% fiber content compared to reference samples. Simultaneously, a slowed degradation was observed for samples with 25% fiber content compared to the reference sample, increasing from 0.2% to 11%.

On the other hand, Karuppasamy et al. [57] conducted a study on unsaturated polyester resin reinforced with powder from *Lansium parasiticum* fruit peels and hemp fibers. Prepared mixtures with different percentages of individual components were tested for physicochemical parameters, including flammability. As a result of the conducted research, a slowed flammability was obtained for samples with 40% hemp fibers and 5% peel powder compared to reference samples.

Kılıç et al. [58] analyzed the basic physicochemical parameters of polypropylene (PP)-based compounds with the addition of hemp stalks (HS) and Maleic Anhydride-grafted Polypropylene (MAPP) modifier. Thermogravimetric analysis (TGA) showed an increase in the amount of residue after burning at 700 °C from 0% to 1.6% for samples with a fiber content of 35%.

The main objective of the present study was to study the effects of two representatives of bast plants found in Eastern Europe, i.e., *Urtica dioica* and *Vitis vinifera*, on the flammability and thermal properties of polylactide. On the other hand, the practical goal of the research study was to test the validity of conducting chemical modification of natural fibers towards lowering the flammability of the biopolymer. Therefore, *Urtica dioica* and *Vitis vinifera* fibers were subjected to two modification processes: mercerization and encapsulation. The fiber fillers prepared in this way were used to obtain polylactide composites using the melt homogenization process, which were subjected to TGA, DSC, MCC, and viscosity tests.

## 2. Materials

To prepare the PLA/natural fiber biocomposites samples, polylactide with the trade name Ingeo™ Biopolymer 3052D was purchased from NatureWorks (Antwerp, Belgium).

We were looking for plants occurring in Poland that belong to the group of plants characterized by the good properties of the fibers obtained from them. Such plants have bast fibers in their structure (they are characterized by simple pits) [59,60,61,62,63,64]. The membranes of such natural fibers become woody with age, moving from cellulose membranes to completely lignified ones. After analyzing Polish vegetation, two species with significantly different structures were selected, i.e., flexible stems of *Urtica dioica* and woody shoots of *Vitis vinifera*.

*Urtica dioica* was collected after the flowering period at the turn of July/August when the plant stems reached their largest dimensions (1–3 m). *Vitis vinifera* shoots were cut during the plant’s dormancy period, i.e., after the leaves fell (October/November). Both types of plants differ quite significantly in terms of structure. *Urtica dioica* is an annual plant, and its stems are poorly developed and flexible. In turn, *Vitis vinifera* is a perennial plant, and its shoots are woody and long-lived. The bark covering the shoots of *Vitis vinifera* peels and falls off.

## 3. Samples Preparation

### 3.1. Natural Fibers’ Treatment

In the first step, leaves were removed from the collected stems and shoots, and then, they were cut into pieces about 2 cm long. Then, both types of plants were dried at 40 °C under a UV lamp ATK UV 350 W (Ataszek, Poznań, Poland) for 6 weeks. In the next stage, the dried plants were ground in a ZAMAK G-16/325 grinder (Zamak Mercator, Skawina, Poland), to obtain short fibers from 0.2 mm in length.

Natural fibers were chemically modified in two different ways. The first method (1M) involved treating dry fibers with a 10% NaOH solution (Merck Life Science, Darmstadt, Germany) for 2 weeks. Then, the fibers were filtered and dried at 40 °C for 4–6 days. The second modification method (2M) was performed using a 10% PLA solution in chloroform (Merck Life Science, Darmstadt, Germany). The chemical treatment and drying time were the same as in the first case.

### 3.2. Biocomposites’ Preparation

**Figure 1:**
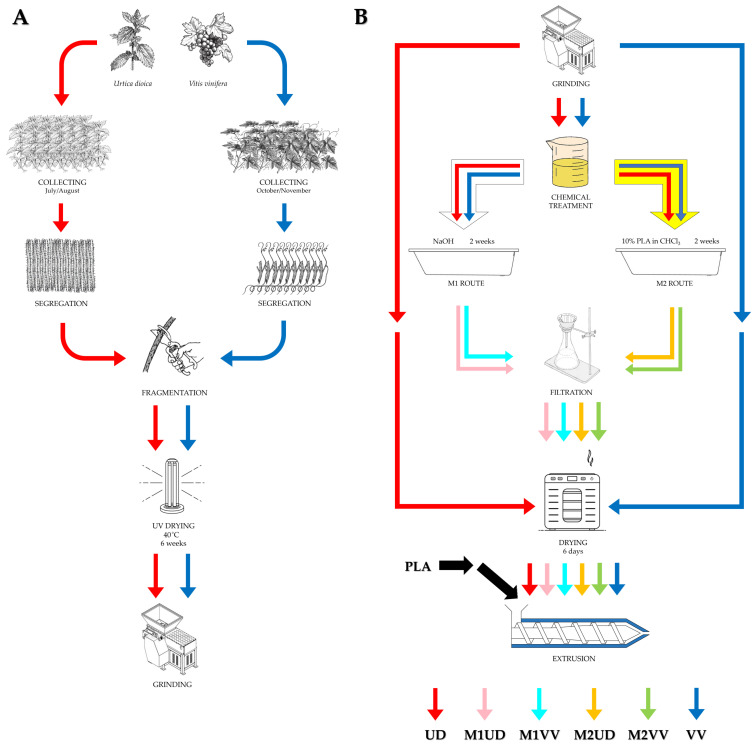
A diagram showing the modification method of natural fibers and the obtainment of polylactide biocomposites that include them. (**A**) raw materials collection; (**B**) samples preparation.

In the preparation process (Figure 1), a processing line consisting of a Brabender DR20 feeder (RHL-Service, Poznań, Poland), twin screw extruder Haake Rheomex OS PTW 16/25 (RHL-Service, Poznań, Poland), cooling bath Zamak W1500 (Zamak Mercator, Skawina, Poland), and pelletizer Zamak G-16/325 (Zamak Mercator, Skawina, Poland) were used.

Using this processing line, PLA, UD, VV, M1UD, M2UD, M1VV, and M2VV composites containing 5, 10, and 15 wt.% of filler were obtained. The processing conditions have been shown in Table 2.

In order to systematize the markings, Table 3 lists all the materials obtained along with the designations used in the study.

## 4. Methods

The viscosity test as a function of temperature was performed using a HAAKE MARS III (RHL-Service, Poznań, Poland) rotational rheometer using Rotor P20 CS L. The test was performed under the following conditions:

- Thermal expansion coefficient of 1.3 μm/°C;
- Humidity of 30%;
- Gap width of 1.0–1.5 mm;
- Temperature range of 180–200 °C;
- Plate-to-plate measurement system;
- Shear rate of 10 1/s;
- Measurement time of 300 s.

The tests were performed in the Control Rate (CR) mode due to the possibility of operating at much higher shear rates, in Pa·s, than in the Control Stress (CS) mode.

Thermogravimetric analysis (TGA) was performed on the NETZSCH TG 209F1 Libra (Netzsch, Cracow, Poland) apparatus. The test was carried out in an oxidizing atmosphere under the following conditions: temperature range from 30 to 600 °C and a heating rate of 10 °C/min. Each sample was tested in an open measuring cell made of Al_2_O_3_, and the weight of the sample was a maximum of 5 mg (measurement error: 0.01 mg).

Micro combustion calorimetry (MCC) was performed using the Fire Testing Technology apparatus (FTT, East Grinstead, Great Britain) according to ASTM D7309. Composite tests were performed in the temperature range of 30–750 °C at a heating rate of 1 °C/s. Before the test, crucibles were weighed, in which samples weighing a maximum of 5 mg were placed (measurement error: 0.01 mg).

The biocomposites with the best thermal and flame retardancy properties were examined for thermal changes using differential scanning calorimetry (DSC) on a Mettler Toledo DSC823e (Mettler Toledo, Warsaw, Poland) apparatus. The test was performed in a nitrogen atmosphere according to the following temperature programs:

- Heating at 25–200 °C at a rate of 10 °C/min;
- Cooling at 200–25 °C at a rate of 10 °C/min;
- Heating at 25–200 °C at a rate of 10 °C/min.

## 5. Results and Discussion

### 5.1. Rheological Tests

**Figure 2:**
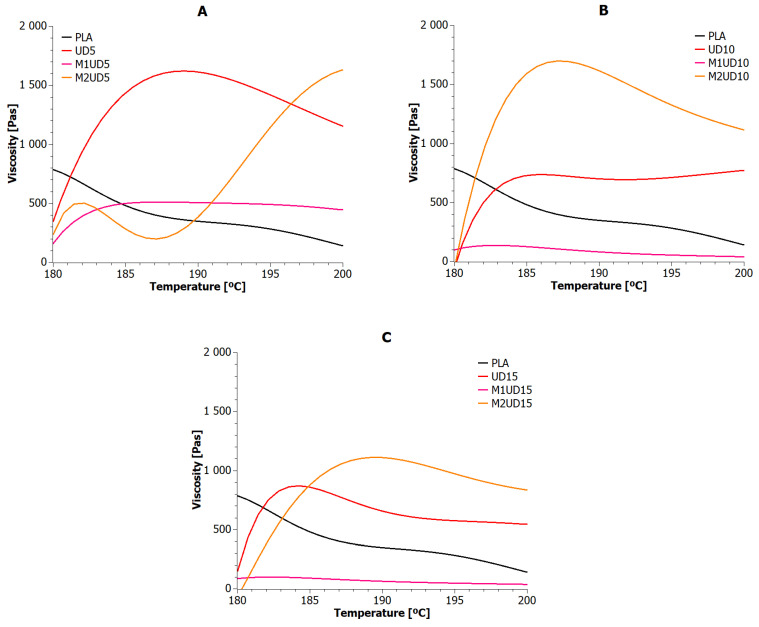
Viscosity versus temperature curves for PLA composites with *Urtica dioica*: (**A**) 5 wt%; (**B**) 10 wt%; and (**C**) 15 wt% of unmodified fibers, fibers modified with route M1, and fibers modified with route M2.

**Figure 3:**
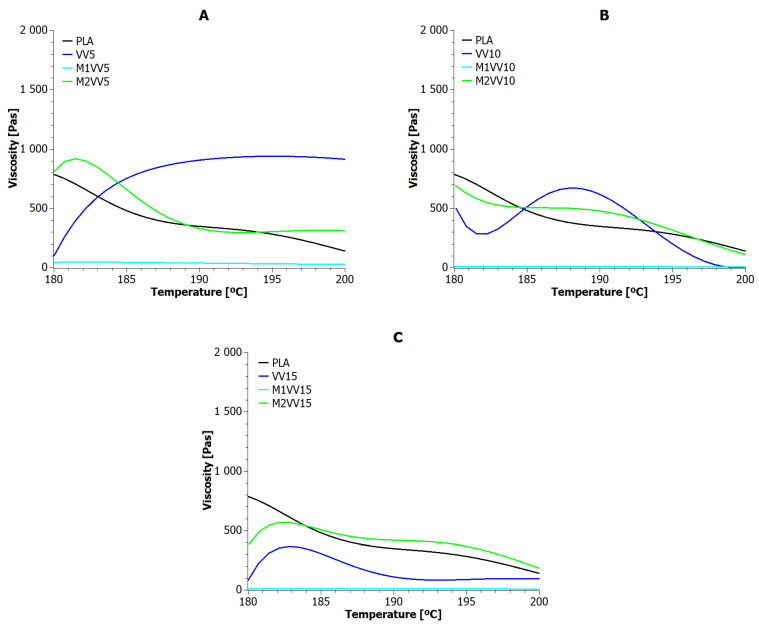
Viscosity versus temperature curves for PLA composites with *Vitis vinifera*: (**A**) 5 wt%; (**B**) 10 wt%; and (**C**) 15 wt% of unmodified fibers, fibers modified with route M1, and fibers modified with route M2.

Thermal stability under the processing conditions of polylactide biocomposites reinforced with natural fibers depends largely on the thermal stability of the fibers themselves. Therefore, it became important to determine the real impact of both modified and unmodified natural fibers on the viscosity of biocomposites while maintaining the temperatures in the extruder’s plasticizing system. The results of these measurements are presented for UD, M1UD, and M2UD composites in Figure 2 and for VV, M1VV, and M2VV in Figure 3. Table 4 lists the viscosity values of biocomposites at the temperatures of individual heating zones.

As the temperature increased, polylactide reached lower and lower dynamic viscosity values. The study did not reach a critical point, beyond which a further increase in temperature would not affect the change in the dynamic viscosity of the reference sample.

In general, the addition of unmodified *Urtica dioica* fibers and those modified with the M2 route resulted in an increase in the viscosity of the composites over the entire measurement range. Nevertheless, the M2 modification allowed to achieve higher mass viscosity than the addition of unmodified fibers. This is because the *Urtica dioica* fibers were additionally encapsulated in a polymer layer before being added to the biopolyester alloy. As shown in Section 5.5, at 190 °C, the biopolymer material was in a molten state and underwent thermo-oxidative degradation. The consequence of further heating of the material under these conditions is the formation of solid char, which causes an increase in viscosity (Figure 2A). The higher the tendency to carbonization, the more pronounced the effect of the changes. The change in viscosity is caused by the disruption of the normal flow of the polylactide, as the fiber and char particles impede the mobility of the chain segments [65]. In turn, the mercerized fibers (M1) plasticized the matrix under processing conditions. This plasticization increased with the amount of these fibers in the biocomposite (see Figure 2A–C). Among the M1UD samples, only the one containing 5 wt% showed results comparable to PLA. It was the first indication that the mercerization of *Urtica dioica* fibers with a NaOH solution removes substances capable of protecting against high temperatures. However, E. Brodos et al. reported that, to improve homogenize natural fibers, the melt viscosity should be 100 Pas. In the presented case, this condition was met only by samples containing mercerized natural fibers [66].

The M2 modification aimed to obtain encapsulated natural fibers, where the layer surrounding the fiber was a biopolymer such as a composite matrix. The rheological measurement results of UD and M2UD composites show that the encapsulation effect is present especially at higher concentrations of *Urtica dioica* fibers. The dynamic viscosity of the biocomposite decreased with the *Urtica dioica* fiber concentration, in each heating zone. However, the increase in temperature along the axis of the screws promoted a slight thickening of the extrudate. Meanwhile, for M2UD composites, an increase in viscosity was visible up to 190 °C. Above this temperature, the higher-filled composites (M2UD10 and M2UD15) showed a trend of lower viscosity, mainly caused by the melting of PLA.

Reinforcing PLA with *Vitis vinifera* is only effective at 5 wt%. Doubling and tripling the concentration of these fibers facilitated the dilution of the composition in almost every heating zone. In this regard, unmodified *Vitis vinifera* fibers appear to be inferior to *Urtica dioica* fibers. A similar conclusion can be reached after analyzing the viscosity values of M1VV composites relative to M1UD. Mercerization of *Vitis vinifera* fibers provides stable, yet lowest, viscosity of samples among all tested materials in all heating zones. Interestingly, stable viscosity values were also observed for M2VV composites, especially the highly filled ones (M2VV10 and M2VV15). This suggests that a characteristic feature of *Vitis vinifera* fibers, regardless of the modification route, is the achievement of stable composition viscosity values under processing conditions. Nevertheless, the tolerance of *Vitis vinifera* fibers tolerate for NaOH solution is much lower than *Urtica dioica* fibers (12, 8, and 6 times lower at 5, 10, and 15 wt% fill-ups respectively). Comparing M2VV and M2UD composites, the effect of modified *Vitis vinifera* fibers on the viscosity of PLA composites is very similar to the effect obtained with *Urtica dioica* fibers modified in the same way.

One best sample can be selected from each of the tested composite types. These include UD5, M1UD5, M2UD10, VV5, M1VV5, and M2VV10. The cohesion of the filling share in biocomposite may suggest that regardless of the type of phloem plant, the highest viscosity values are obtained with the same filling for a given modification route.

### 5.2. Thermogravimetric Analysis (TGA)

**Figure 4:**
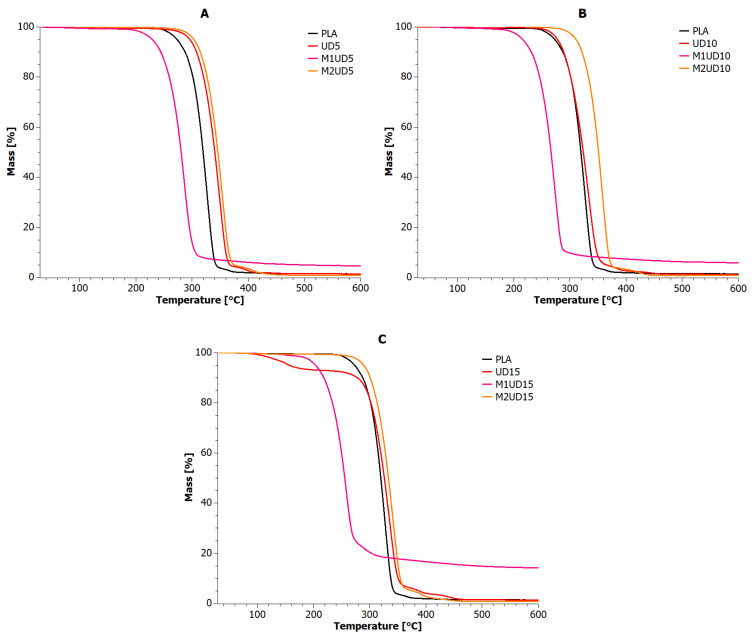
TGA curves for PLA composites with *Urtica dioica*: (**A**) 5 wt%; (**B**) 10 wt%; and (**C**) 15 wt% of unmodified fibers, fibers modified with route M1, and fibers modified with route M2.

**Figure 5:**
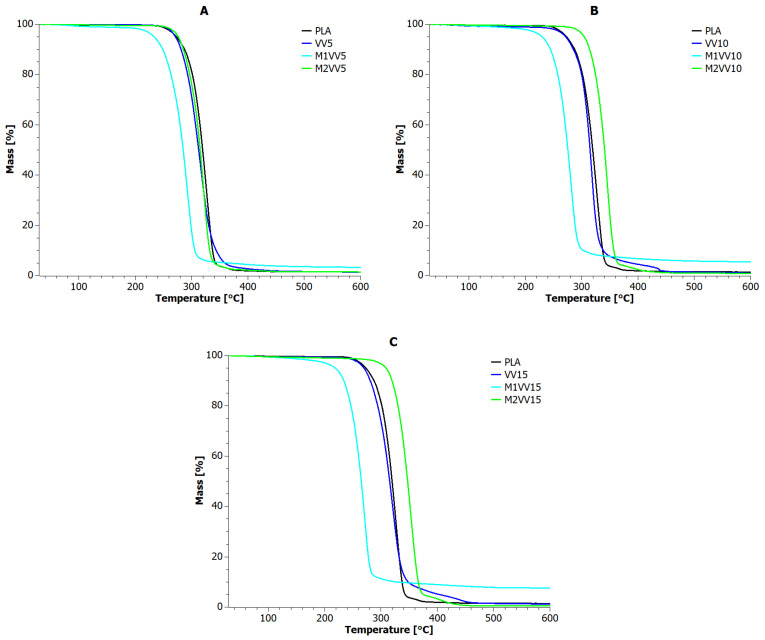
TGA curves for PLA composites with *Vitis vinifera*: (**A**) 5 wt%; (**B**) 10 wt%; and (**C**) 15 wt% of unmodified fibers, fibers modified with route M1, and fibers modified with route M2.

A dynamic thermogravimetric study (TGA) was carried out to observe changes in the mass of samples of the manufactured PLA composites reinforced with *Urtica dioica* and *Vitis vinifera* fibers. Figure 4 and Figure 5 show the changes in sample mass as a function of temperature for UD and VV composites, respectively. Table 5 contains TGA indices determined for PLA composites based on these curves.

The reference sample was characterized by two stages of decomposition, i.e., the first main stage in the range of up to 350 °C and the second in the narrow range of 350–380 °C, ultimately reaching 1.44% of residue. All M1UD and M1VV composites had lower thermal stability than PLA, and the increase in filling in these samples was directly proportional to the carbon level achieved at 600 °C. The use of mercerized natural fibers also resulted in the disappearance of the second stage of decomposition in the range of 350–380 °C. This means that the modification of natural fibers according to the M1 route allows to obtain a filler with the ability to form char at temperatures lower by 45–75 °C (M1UD) and 40–60 °C (M1VV), respectively, than in the case of PLA.

When analyzing the UD and VV curves, it should be mentioned that most materials decompose in two stages. The char afterburning for all UD samples occurs from 375 to 410 °C. Therefore, 30 °C later than for PLA. Even better results were obtained for samples VV10 and VV15. Here, the second stage of decomposition began only at 400 °C and ended at 475 °C. This is over 50° difference compared to PLA.

The only anomalies were observed for samples UD15 and VV5. The former was characterized by a four-stage decomposition process, while the latter had only one stage. However, the most important observation is the fact that all samples containing unmodified *Vitis vinifera* fibers showed on average 13 °C lower thermal stability compared to PLA. In turn, the biocomposite containing 10 wt% of *Urtica dioica* fibers did not show any significant changes in the discussed course of the TGA curve (Figure 4B). Only the addition of 5% *Urtica dioica* to PLA (Figure 4A) was able to improve thermal stability by more than 15 °C.

The most promising type of modification turned out to be the M2 route. Almost all biocomposite samples containing natural fibers modified according to the M2 route were characterized by improved thermal properties compared to PLA. The only sample that did not meet all the necessary criteria was M2VV5. This may mean that, in the case of *Vitis vinifera* fibers, filling the matrix with their content above 5% by mass may provide an effective thermal stability effect (Figure 5B,C).

It is also worth noting the relationship between the residue values at 600 °C and the values of thermogravimetric indicators. Specifically, the lower the solid residue value, the higher the thermal stability of biocomposites. The thermal stability of M2UD and M2VV samples increased on average by nearly 18 °C compared to PLA. All M2UD and M2VV samples were also characterized by a two-stage degradation mechanism. However, it should be noted that, for M2UD composites, the second stage of decomposition occurred in the range of 390–450 °C, and for M2VV composites, in the range of 375–400 °C. Thus, M2UD biocomposites had a charring effect in the range of VV samples, and, conversely, M2VV samples were charred like UD composites.

### 5.3. Micro Combustion Calorimetry (MCC)

**Figure 6:**
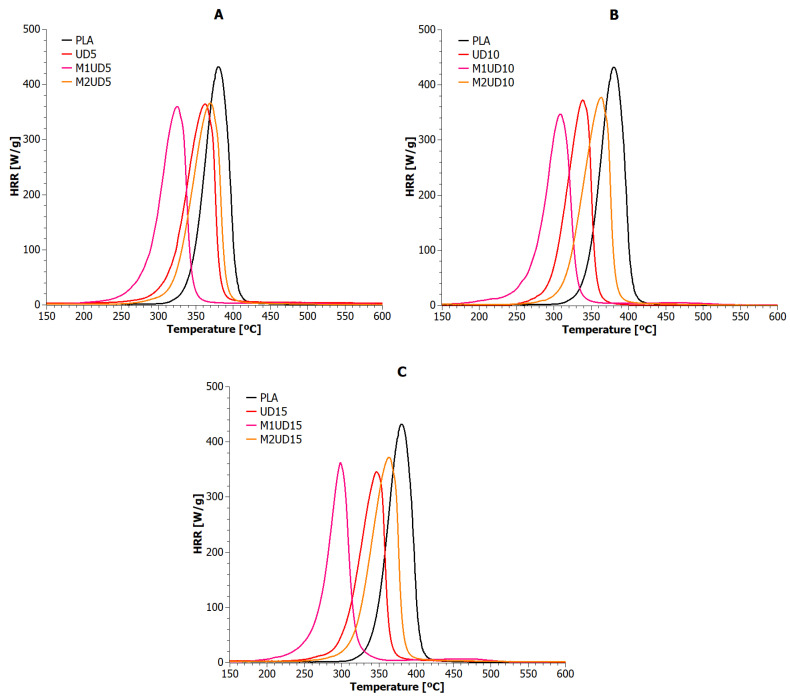
Heat release rate versus temperature for PLA composites with *Urtica dioica*: (**A**) 5 wt%; (**B**) 10 wt%; and (**C**) 15 wt% of unmodified fibers, fibers modified with route M1, and fibers modified with route M2.

**Figure 7:**
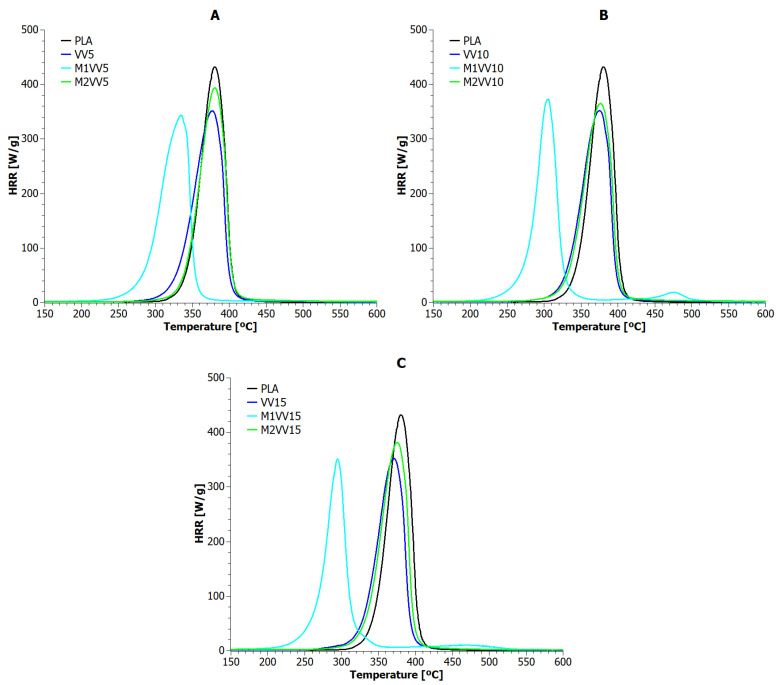
Heat release rate versus temperature for PLA composites with *Vitis vinifera*: (**A**) 5 wt%; (**B**) 10 wt%; and (**C**) 15 wt% of unmodified fibers, fibers modified with route M1, and fibers modified with route M2.

Microcalorimetric testing allowed for the observation of the heat release rate (HRR) as a function of time and temperature for polylactide composites reinforced with natural fibers. The obtained results are presented in Figure 6 and Figure 7 and Table 6.

The highest values of heat release rate were achieved by the reference sample (432 W/g at 380 °C). All polylactide compositions reinforced with natural fibers (unmodified and modified) achieved lower values for the maximum heat release rate (PHRR). In this regard, the tested biomaterials can be ranked as follows: UD15, M1UD5, M1UD10, all VV samples, M1VV5, M1VV15 >> UD5, UD10, M1UD15, all M2UD samples, M1VV10, M2VV10 >>> M2VV15 >>>> M2VV5. In addition to the PHHR analysis, it is also important to compare indicators such as Time To Ignition (TTI), Time Out Flame (TOF), and sample combustion time (Table 6). Also, all composite samples ignited faster than PLA, including the fastest (~80 s faster) samples M1UD and M1VV containing 10 and 15 wt% mercerized fibers (Figure 6B,C and Figure 7B,C). The same samples were also extinguished over 1.5 min faster than pure PLA. Therefore, these samples burned for the shortest time (less than a minute). These observations show one common relationship: the higher the PHRR reduction compared to the reference sample, the shorter the combustion time. According to this hypothesis, the highest flammability reduction was achieved for composites containing mercerized natural fibers. These biocomposites had the lowest combustion times. The influence of the share of these fibers on the obtained values depended on the type of biofillers used. If mercerized *Urtica dioica* fibers were used, the best effect was obtained at 10 wt% filling (Figure 6A), and if *Vitis vinifera* fibers were used, it was achieved at 5 and 15 wt% (Figure 7A,C).

Intermediate results were obtained after using unmodified fibers. VV and UD composites burned either longer or identically to PLA (depending on the share in the matrix), achieving an approximately 17% reduction in PHRR. It is important that, regardless of the share of *Vitis vinifera* fibers used, an identical level of PHRR was achieved. The amount of *Vitis vinifera* fibers had only a real impact on TTI, TOF, and combustion time. Unfortunately, such an effect was not observed in samples containing *Urtica dioica* fibers. Unmodified *Urtica dioica* fibers were more responsible for the heterogeneity of flammability characteristics.

The weakest flammability reduction was obtained for composites modified by the M2 route (~14% for M2UD and ~12% for M2VV). Here, on the contrary, both types of composites had the TTI and TOF points most similar to PLA. These samples even burned several seconds longer than the pure biopolymer matrix. The observed effect is opposite to that observed in the literature for synthetic materials [20,21,23], where the consequence of lowering the PHRR point is the extension of the combustion time of polymeric materials. In the case of the discussed results, the effect is the opposite, i.e., the consequence of lowering PHRR is also the shortening of the combustion time. The observed impact of fibers modified by the M2 route on the real values of flammability indicators was the same as when discussing composites containing unmodified fibers. Taking into account all the indicated flammability characteristics, the following samples with the best results were selected from each type of biocomposite: UD5, M1UD5, M2UD5, VV10, M1VV5, and M2VV10. As observed in most cases, only 5 wt% of natural fibers allow the achievement of the best effects by reducing the flammability of PLA.

### 5.4. TGA vs. MCC

Based on the ASTM D7309 standard, additional flammability indicators were also determined, such as fire growth capacity (FGC) and heat release capacity (η_c_). The results of these indicators for the tested materials are summarized in Table 7.

The first observation was that the addition of 5 wt% of both unmodified *Urtica dioica* and *Vitis vinifera* fibers as well as M2 route-modified *Vitis vinifera* fibers to PLA increased the total heat released (THR, known as h_c_). In all other cases, THR values were obtained lower than those recorded for the reference sample. Moreover, with the increase in the share of mercerized fibers, there was a successive decrease in the ability of these samples to release heat with a simultaneous decrease in FGC, compared to pure PLA. Therefore, a certain relationship between THR and FGC can be attributed to the influence of mercerized fibers on the flammability of PLA composites, i.e., the lower the THR (h_c_) values, the lower the FGC and vice versa. This result confirms the findings of a previous report indicating a significant impact of M1 modification on the flammability of PLA biocomposites [23]. This means that the modification of natural fibers becomes necessary if we want to reduce flammability. Composites containing unmodified *Vitis vinifera* fibers (VV) and M2UD samples were not able to achieve even half the same results. Meanwhile, among the M2VV composites, only those containing 5 and 10 wt% reached the level of THR and FGC indicators corresponding to mercerized samples.

Mercerization of natural fibers was intended to expose the inner part of the bast fiber. Thanks to the results obtained, it is known that, regardless of the type of starting plant used, its modification via the M1 route produces a very similar effect. Exposing inaccessible parts of the fibers first provides the fibers with the ability to carbonize more quickly, shifting thermal stability towards lower temperatures. This faster carbonization leads to the formation of a barrier that protects the matrix from premature decomposition. Completely environmentally friendly combustion inhibitors include flame retardants that promote the formation of a carbonized product called coke when the polymer burns. Therefore, it can be safely said that the M1 modification leads to obtaining coking bio-flame retardant. Flame retardants that catalyze the formation of coke in the combustion process are a good alternative to phosphorus compounds popular in Europe. Taking into account the existence of a clear relationship between the carbonization efficiency and the fire resistance of the biomaterial, this research study was aimed at intensifying the transformation of biopolymers into coke during combustion processes.

**Figure 8:**
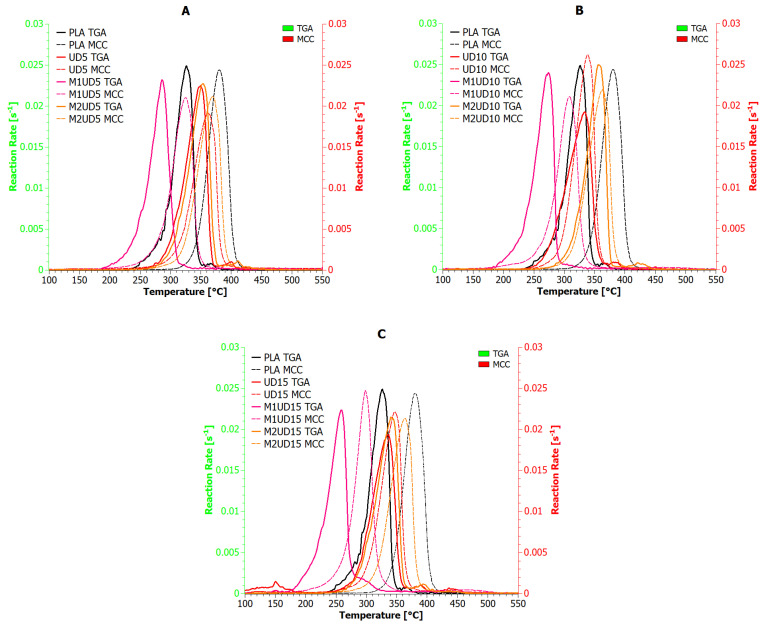
Reaction rate for polylactide composites with *Urtica dioica*: (**A**) 5 wt%; (**B**) 10 wt%; and (**C**) 15 wt% of unmodified fibers, fibers modified with route M1, and fibers modified with route M2.

**Figure 9:**
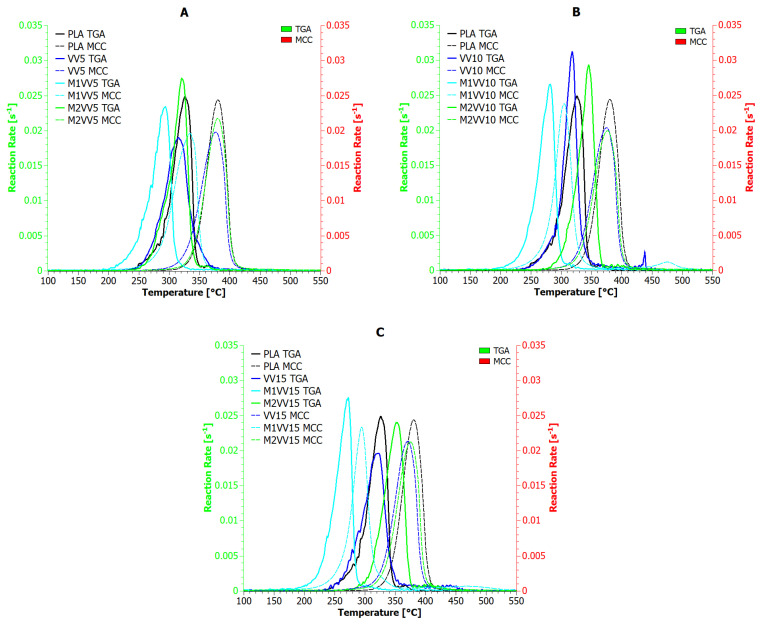
Reaction rate for polylactide composites with *Vitis vinifera*: (**A**) 5 wt%; (**B**) 10 wt%; and (**C**) 15 wt% of unmodified fibers, fibers modified with route M1, and fibers modified with route M2.

Figure 8 and Figure 9 show the results of the reaction rate (RR) of decomposition determined based on TGA (green *X*-axis) and MCC (red *X*-axis) measurements for composites containing *Urtica dioica* and *Vitis vinifera* fibers, respectively. Based on the presented analysis, it can be concluded that the rate of the composite decomposition reaction depends on the rate of the combustion process. For example, for UD, M1UD, and M2UD composites, the difference between the maximum points of the decomposition reaction rate for the TGA and MCC curves is identical at 5 and 15 wt% (Figure 8A,C and Figure 9A,C), and the smallest for composites filled with 10 wt%. In turn, for the VV, M1VV, and M2VV composites, as the share of fibers in the composite increases, the differences between the maximum points of the decomposition reaction rate for the TGA and MCC curves decrease.

According to literature reports on research on the kinetics of biomaterial decomposition, as the combustion rate increases, the temperatures at which the maximum RR occurs also increase [67,68,69]. Biocomposites containing mercerized fibers at a heating rate of 10 °C/min reach a maximum decomposition rate above 270 °C. While increasing the heating rate by a factor of six shifts this point by an average of 30 °C to the right. Encapsulation of *Urtica dioica* fibers according to the M2 route produces different results than their mercerization. Because the decomposition rate of M2UD composites was the least dependent on the biomaterial heating rate, reducing the indicated shift by half. An increasing carbonaceous charring decreases the released gas and condensed phase and increases the residue.

To finally compare the effect of *Urtica dioica* and *Vitis vinifera* fibers on the thermal stability and flammability of polylactide composites, the Overall Thermal Stabilization Effect (OSE) and Overall Flammability Effect (OFE) values were determined using the following Equations (1) and (2):

(1) $$OSE=\sum_{T=150}^{600}\left(\left(mass\;percent\;of\;polymer\;material_{T}\right)-\left(mass\;percent\;of\;polylactide_{T}\right)\right)$$

(2) $$OFE=\sum_{T=150}^{600}\left(\left(HRR\;percent\;of\;polymer\;material_{T}\right)-\left(HRR\;percent\;of\;polylactide_{T}\right)\right)$$

**Figure 10:**
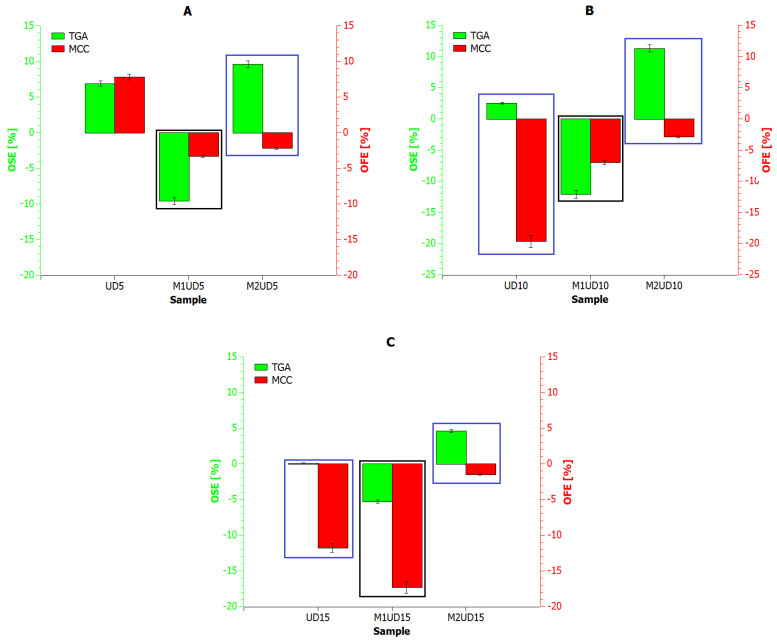
Overall Thermal Stabilization Effect (OSE) and Overall Flammability Effect (OFE) for PLA composites with modified and pure *Urtica dioica* fibers: (**A**) 5 wt%; (**B**) 10 wt%; and (**C**) 15 wt% of unmodified fibers, fibers modified with route M1, and fibers modified with route M2.

**Figure 11:**
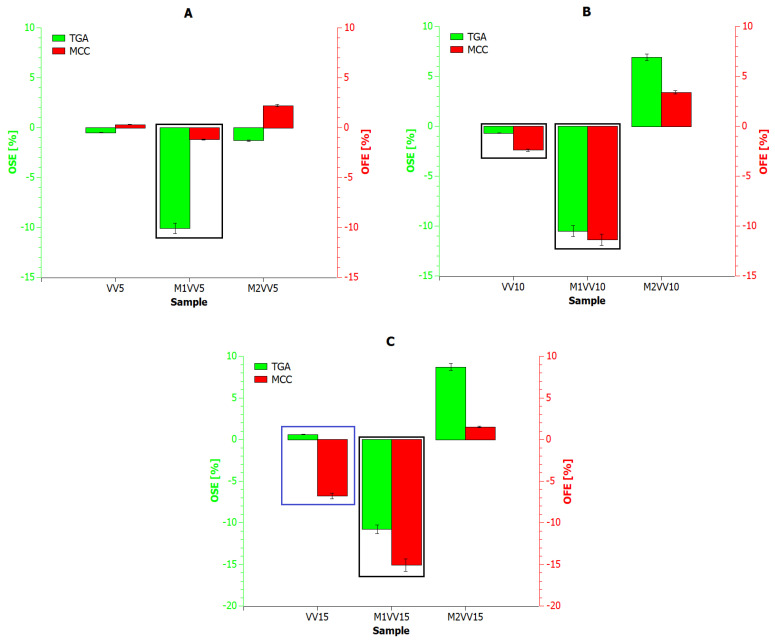
Overall Thermal Stabilization Effect (OSE) and Overall Flammability Effect (OFE) for PLA composites with modified and pure *Vitis vinifera* fibers: (**A**) 5 wt%; (**B**) 10 wt%; and (**C**) 15 wt% of unmodified fibers, fibers modified with route M1, and fibers modified with route M2.

The obtained values of the OSE and OFE indicators related to the PLA reference sample are summarized in Figure 10 and Figure 11. The blue frame marks the biocomposites in which thermal stability was improved and flammability was reduced compared to PLA. However, a black frame marks composites which, due to their deteriorated thermal stability, show a faster charring effect, which reduces flammability compared to PLA.

The first criterion is met by samples modified according to the M2 route, i.e., M2UD, and composites reinforced with 10 and 15 wt% of unmodified *Urtica dioica* fibers (Figure 10B,C) and 15 wt% of pure *Vitis vinifera* fibers (Figure 11C). Meanwhile, the second criterion is met primarily by samples containing mercerized fibers, which is consistent with previous research results. Mercerization is helpful in removing some amount of lignin and impurities, which cover the fiber surface. Also, it is one of the best ways to improve fiber adhesion to polylactide. Bast fibers have a hydrophilic nature, and alkali treatment is one of the best solutions that removes fatty deposits like the waxy and oily contents from the fiber. Furthermore, the mercerization changes the color from brown to dark brown, which shows that the anticipated modification has been achieved [14,24,25]. As previously proven, M1UD and M1VV composites were characterized by significantly reduced viscosity at processing temperatures. Alkali treatment results in a rough fiber surface and enhances the fiber surface area, leading to excellent interlocking with the PLA. Better compatibility between the dispersed phase and the matrix promoted the plasticization of the mass in the molten state [7,13,25,28].

PLA composites with mercerized fibers without fats decomposed only in one stage. The presence of waxy contents in the fiber, such as lignin, hemicellulose, and pectin, on the one hand, contributed to increasing thermal stability and decomposition stages, but at the same time led to the formation of less valuable solid chars during decomposition. Taking into account all the above factors, composites containing 10 wt% mercerized fibers were selected for DSC analysis.

### 5.5. Differential Scanning Calorimetry (DSC)

**Figure 12:**
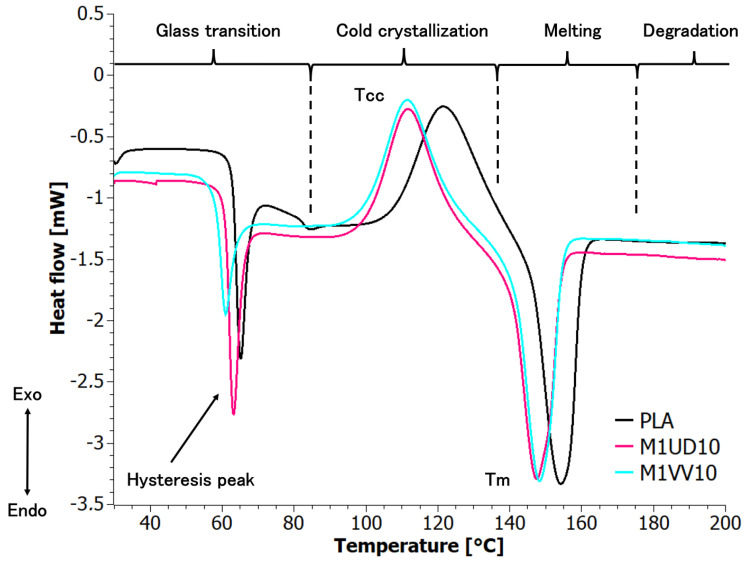
DSC curves for selected polylactide composites.

Figure 12 shows the DSC plot for the previously selected PLA composites and reference sample. Based on graphical interpretation, DSC indicators were determined and are presented in Table 8. The degree of crystallinity was also calculated using Equation (3) [70,71,72]:

(3) $$X_{C}=\frac{\Delta H_{m}-\Delta H_{CC}}{\left(1-\omega \right)\cdot \Delta H^{o}}$$

where

Δ*H_m_*—change in melting enthalpy of a polymer sample;

Δ*H_CC_*—change in enthalpy of cold crystallization;

Δ*H^o^*—change in melting enthalpy of a completely crystalline polymer equal to 93 J/g [73];

*ω*—filler content.

The stages of phase transformations are conventionally marked in Figure 12: glass transition, cold crystallization, melting, and degradation. The glass transition stage was characterized by a visible hysteresis peak typical of polylactide. This condition occurs whenever the sample is heated at a different rate than it was previously cooled. A further consequence of this phenomenon is the occurrence of the cold crystallization effect. Analyzing the DSC curves in this temperature range (30–85 °C), it was noted that the presence of the modified fibers in each form slightly reduced the glass transition temperature (*T_g_*) of the composites compared to the PLA sample. The content of filler in the PLA resulted in two- and threefold increases in melting enthalpy (Δ*H_m_*), respectively. The reason was that the addition of *Urtica dioica* and *Vitis vinifera* hindered the interfacial transformation from the glassy state to the plastic state and was documented by the need to supply more heat to reach the transition point between these two states.

The addition of mercerized fillers to PLA resulted in a shift of cold crystallization (*T_CC_*) and crystallization (*T_c_*) temperatures towards lower values. Further heating of the materials caused one-stage melting of the analyzed biocomposites.

## 6. Conclusions

*Urtica dioica* possesses thorns called trichomes which contain histamine, acetylcholine, and formic acid [74,75]. Thanks to these compounds, *Urtica dioica* has primarily antioxidative properties [76,77], which makes it attractive for use as a natural antioxidant.

Another natural precursor from the same species of bast plants is *Vitis vinifera*. *Vitis vinifera* owes its antioxidant properties to phenolic compounds such as resveratrol [78].

The above research allowed us to discover a new feature of these fibers. Mercerized *Urtica dioica* and *Vitis vinifera* fibers are capable of generating a large portion of solid residue at lower temperatures than when using these unmodified or encapsulated fibers. Chemical modification primarily removes fatty compounds responsible for the ability to absorb moisture during the processing process. Thus, chemical modification helps to increase the hydrophobicity of the surface of natural fibers and, consequently, compatibility with the PLA matrix [25,79,80,81,82]. This is confirmed, among others, by results of changes in viscosity as a function of temperature, where fibers without additional ingredients better wetted the biopolymer surface, leading to its plasticization. This is because the consequence of converting hydroxyl groups to Cellulose–Na linkages is the shortening of fiber length by up to 18% [83]. Moreover, the cellulose molecules become more exposed on the surface, which promotes the improved adhesion of the fibers to the polylactide matrix due to a greater number of possible reaction sites [84]. Most importantly, the addition of modified fibers does not change the characteristics of the phase transformations of the bio-matrix but affects the scope of key endothermic and exothermic changes during heating.

Modification with the M1 route allows for a flammability reduction of 20% compared to PLA, which was not achieved even when the fibers were enclosed in a capsule made of the same polymer. The share of bast fibers also plays a particular role in the thermal and flammability properties of the obtained composite. A fiber content of 5 wt% is insufficient to exceed the rates defined for PLA, and 15 wt% generally leads to results worse or similar to those obtained with 5 wt% fiber content. Therefore, the most favorable results of changes in thermal stability and flammability were obtained for composites containing 10 wt% mercerized natural fibers.

## Figures and Tables

**Table 1:** Summary of the occurrences of combination of selected keywords for the Scopus database.

Selected Keywords	Number of Phrases in Scopus
Step no 1	
natural fibers, thermal properties	5873
natural fibers, flammability	383
natural fibers, biopolymer	1561
natural fibers, *Urtica dioica*	35
natural fibers, *Vitis vinifera*	16
Step no 2	
natural fibers, biopolymer, thermal properties	266
natural fibers, biopolymer, flammability	19

**Table 2:** Processing conditions.

Extruder							
Temperature [°C]	Heating zones						
	1	2	3	4	5	6	Die
	180	180	185	190	195	200	200
Degassing	-	-	-	-	Yes	-	-
Screws speed [rpm]	150						
Feed capacity [%]	5						
Cooling bath							
Temperature [°C]	20						

**Table 3:** The list of investigated samples.

No.	Type of Fiber	Route of Modification	Name of Matrix	Filling Percentage [%]	Composite Designation	Color on Figures
1	-	-	PLA	-	PLA	Black
2	*Urtica* *dioica*	-		5	UD5	Red
3				10	UD10	
4				15	UD15	
5		M1		5	M1UD5	Pink
6				10	M1UD10	
7				15	M1UD15	
8		M2		5	M2UD5	Orange
9				10	M2UD10	
10				15	M2UD15	
11	*Vitis* *vinifera*	-		5	VV5	Blue
12				10	VV10	
13				15	VV15	
14		M1		5	M1VV5	Cyan
15				10	M1VV10	
16				15	M1VV15	
17		M2		5	M2VV5	Green
18				10	M2VV10	
19				15	M2VV15	

**Table 4:** Viscosity values read at temperatures corresponding to the heating zones (plasticizing system) of the extruder.

Sample	η at 180 °C [Pas]	η at 185 °C [Pas]	η at 190 °C [Pas]	η at 195 °C [Pas]	η at 200 °C [Pas]
PLA	786.6	485.7	349.8	285.6	145.2
UD5	281.3	1412.9	1616.8	1432.3	1167.4
UD10	152.5	725.5	702.6	709.5	769.0
UD15	152.9	860.5	659.4	576.3	545.7
M1UD5	156.8	499.3	507.0	491.5	446.2
M1UD10	98.6	127.0	82.5	53.0	40.9
M1UD15	87.3	90.7	64.4	48.6	38.1
M2UD5	239.9	282.8	381.3	1138.9	1629.9
M2UD10	354.5	1592.2	1617.4	1327.5	1116.0
M2UD15	96.5	878.7	1110.6	974.3	836.3
VV5	103.0	753.6	906.8	939.8	914.9
VV10	347.8	454.1	649.1	258.3	7.8
VV15	223.9	308.4	112.0	88.4	93.9
M1VV5	45.3	46.3	39.9	33.1	27.2
M1VV10	11.8	11.7	10.4	9.2	7.9
M1VV15	11.9	11.9	11.5	10.2	8.4
M2VV5	714.1	628.3	330.6	303.6	313.1
M2VV10	693.3	509.2	488.4	345.8	111.6
M2VV15	386.5	529.6	422.9	368.0	185.1

**Table 5:** TGA indices determined for PLA composites based on Figure 4 and Figure 5.

Sample	T_5%_ [°C]	T_10%_ [°C]	T_20%_ [°C]	T_50%_ [°C]	T_MAX_ [°C]	Residue at 600 °C [%]
PLA	273	287	301	319	326	1.44
UD5	296	308	320	340	349	1.40
UD10	278	288	302	323	334	1.09
UD15	156	278	302	325	334	1.34
M1UD5	229	244	259	280	286	4.60
M1UD10	216	231	246	266	275	5.83
M1UD15	204	218	233	255	260	14.24
M2UD5	303	314	326	345	353	0.86
M2UD10	311	321	333	350	357	0.79
M2UD15	289	301	313	333	342	0.89
VV5	272	281	292	312	316	1.38
VV10	272	285	300	315	319	1.06
VV15	270	280	294	315	321	1.15
M1VV5	234	249	263	285	293	3.26
M1VV10	230	244	257	275	282	5.45
M1VV15	218	234	246	265	272	7.55
M2VV5	276	285	296	315	321	1.42
M2VV10	305	314	324	340	345	0.87
M2VV15	309	319	330	345	353	0.50

**Table 6:** The list of flammability indicators determined based on Figure 6 and Figure 7.

Sample	PHRR [W/g]	TTI		TOF		Combustion Time [s]
		[s]	[°C]	[s]	[°C]	
PLA	432	303	342	370	407	67
UD5	364	265	311	340	385	75
UD10	372	245	294	312	358	67
UD15	346	250	301	315	365	65
M1UD5	360	239	278	309	347	70
M1UD10	347	227	268	295	335	68
M1UD15	362	216	265	271	320	55
M2UD5	366	277	318	353	392	76
M2UD10	377	277	312	349	382	72
M2UD15	372	278	315	348	382	70
VV5	352	275	327	349	400	74
VV10	352	296	327	371	400	75
VV15	352	282	327	353	396	71
M1VV5	344	233	280	309	355	76
M1VV10	373	227	273	283	328	56
M1VV15	351	223	259	280	316	57
M2VV5	394	301	338	373	408	72
M2VV10	365	292	336	364	405	72
M2VV15	382	283	332	354	401	71

**Table 7:** Flammability factors for polylactide composites calculated according to ASTM D7309 standard, where FGC—fire growth capacity of sample; h_c_—specific heat release in the test; η_c_—heat release capacity; T_5%_—temperature in the test at which 5% of h_c_ has been released measured at a heating rate β = 1 K/s; and T_95%_—temperature in the test at 95% of h_c_ has been released measured at a heating rate, β = 1 K/s.

Sample	Q_max_ [W/g]	T_5%_		T_95%_		FGC [J/gK]	η_c_ [J/gK]	h_c_ [kJ/g]
		[s]	[K]	[s]	[K]			
PLA	432	301	614	365	673	353.93	481.11	17.70
UD5	364	248	567	419	743	179.33	401.22	19.07
UD10	372	247	569	304	623	318.16	409.53	14.21
UD15	346	229	552	320	644	231.79	374.76	15.61
M1UD5	360	222	534	316	629	252.18	395.63	17.11
M1UD10	347	200	514	307	623	227.03	381.93	16.45
M1UD15	362	190	513	287	611	217.45	395.15	14.64
M2UD5	366	274	588	349	661	297.32	399.51	17.30
M2UD10	377	274	582	346	652	305.63	421.16	17.18
M2UD15	372	270	580	353	661	277.63	413.68	17.44
VV5	352	270	595	349	673	286.83	382.91	17.75
VV10	352	294	598	370	672	289.92	392.66	17.28
VV15	352	275	593	352	668	274.86	388.10	16.49
M1VV5	344	225	545	320	641	251.61	376.71	17.49
M1VV10	373	210	529	417	742	141.55	410.61	15.68
M1VV15	351	207	517	434	748	133.55	390.93	15.02
M2VV5	394	298	609	390	703	249.33	439.94	18.09
M2VV10	365	280	596	388	707	226.29	402.00	18.31
M2VV15	382	273	595	354	674	288.38	417.83	17.96

**Table 8:** DSC indicators: melting temperature (T_m_), melting enthalpy (ΔH_m_), degree of crystallinity (X_c_),crystallization temperature (T_c_), cold crystallization temperature (T_cc_), and glass transition temperature (T_g_).

Sample	T_g_ [°C]	T_cc_ [°C]	T_m_ [°C]	∆H_m_ [J/g]	T_c_ [°C]	X_c_ [%]
PLA	63	122	154	10	119	11
M1UD10	58	111	147	21	115	23
M1VV10	57	111	148	29	113	32

## Data Availability

The data are contained within the article.
